# Group XIV C-type lectins: emerging targets in tumor angiogenesis

**DOI:** 10.1007/s10456-024-09907-x

**Published:** 2024-03-12

**Authors:** Elliott J. Yee, Isaac Vigil, Yi Sun, Robert J. Torphy, Richard D. Schulick, Yuwen Zhu

**Affiliations:** 1 Department of Surgery, University of Colorado Anschutz Medical Campus, 12800 E 19th Avenue, RC1-North, P18-8116, Aurora, CO 80045 USA; 2 Department of Surgery, Memorial Sloan Kettering Cancer Center, New York City, NY USA

**Keywords:** Tumor angiogenesis, C-type lectin, Vessel normalization, Immunotherapy

## Abstract

C-type lectins, distinguished by a C-type lectin binding domain (CTLD), are an evolutionarily conserved superfamily of glycoproteins that are implicated in a broad range of physiologic processes. The group XIV subfamily of CTLDs are comprised of CD93, CD248/endosialin, CLEC14a, and thrombomodulin/CD141, and have important roles in creating and maintaining blood vessels, organizing extracellular matrix, and balancing pro- and anti-coagulative processes. As such, dysregulation in the expression and downstream signaling pathways of these proteins often lead to clinically relevant pathology. Recently, group XIV CTLDs have been shown to play significant roles in cancer progression, namely tumor angiogenesis and metastatic dissemination. Interest in therapeutically targeting tumor vasculature is increasing and the search for novel angiogenic targets is ongoing. Group XIV CTLDs have emerged as key moderators of tumor angiogenesis and metastasis, thus offering substantial therapeutic promise for the clinic. Herein, we review our current knowledge of group XIV CTLDs, discuss each’s role in malignancy and associated potential therapeutic avenues, briefly discuss group XIV CTLDs in the context of two other relevant lectin families, and offer future direction in further elucidating mechanisms by which these proteins function and facilitate tumor growth.

## Introduction

Pathologic angiogenesis is a hallmark of malignant tumors [1]. Growth of the primary tumor and metastatic progression depend on direct connection to the circulatory system for oxygen and nutrients [2]. Tumors can either co-opt existing blood vessels or create new ones via neoangiogenesis [3]. These new vessels, however, are often leaky, chaotic, and dysfunctional, contributing to the characteristic aberrant metabolic microenvironment defined by acidosis, hypoxia, and deranged glucose metabolism [4]. Yet, these defining dysregulated features also seem to drive a tumor’s ability to persist in and evade our otherwise hostile environment.

For over nearly half a century, targeting tumor angiogenesis to disrupt a tumor’s nutrient supply has been explored as an anti-tumor strategy [5]. Recently, interest in the mechanisms responsible for tumor-associated angiogenesis has been reinvigorated by the concept of normalizing the dysregulated vasculature of the tumor microenvironment (TME) as means to facilitate host immune response and influx of systemic therapies. The moderate success of anti-vascular endothelial growth factor (VEGF) targeting in metastatic colorectal cancer (CRC) has demonstrated the efficacy of anti-angiogenic therapies in real-world experience [6–8]. However, the realities of therapy resistance and limited applicability to a majority of solid organ malignancies make evident our nascent understanding of the mechanisms underlying tumor angiogenesis and how to target them [9].

C-type lectins are an evolutionarily conserved protein superfamily that boasts an extensive breadth of domain architecture, signaling pathways, and function [10]. Defined by their hallmark C-type lectin binding domain (a double-looped, two stranded antiparallel beta-sheet) aka a CTLD, this family of over 1000 mammalian members have been classified into 17 subfamilies, grouped by structural and functional similarities [11]. Group XIV proteins, including CD93 (C1qRp), thrombomodulin (CD141, TM), CD248/endosialin (tumor endothelial marker 1/TEM1), and C-type lectin family member 14a (CLEC14a), are a subfamily of transmembrane CTLDs with intimate involvement in physiologic angiogenesis, cell adhesion, and regulation of inflammation. Their importance to normal physiologic processes has been paradoxically highlighted by group XIV CTLDs’ emerging roles as significant drivers of tumor angiogenesis and metastatic dissemination [12, 13]. Further, preclinical studies demonstrate that the therapeutic targeting of group XIV CTLDs result in significant anti-tumor benefit, some currently being tested in the clinic.

Given the recent success in targeting the tumor vasculature via the VEGF pathway and the emerging role of CTLD proteins in cancer angiogenesis and progression [14–16], we aim to give a brief overview of the Group XIV CTLD members within the larger context of C-type lectins, review our current understanding of each group XIV protein’s role in normal biology as well as tumorigenesis, from a mechanistic and therapeutic perspective, provide a brief review of group XIV CTLDs in the context of other well-described lectins, and offer potential future direction in expanding our knowledge of the mechanisms behind CTLDs and the avenues to target them.

### Group XIV C-type lectins: an overview

CTLDs are an evolutionarily conserved superfamily of proteins with vast variability in structure, binding ligands, and function [11]. The structural hallmark of CTLDs is a double-looped, two stranded antiparallel *β*-sheet binding site known as the C-type lectin binding domain. Significant sequence variability in CTLD structure in addition to unique domain architecture have enabled classification of 17 known subfamilies of mammalian CTLDs [17]. Originally, thought to strictly bind carbohydrates via a calcium dependent process, it is now known that many human CTLD members do not bind carbohydrates nor require calcium to bind its ligands including proteins, lipids, and inorganic molecules [17]. The diversity in binding ligands for this superfamily is mirrored by their diversity of function.

**Fig. 1:**
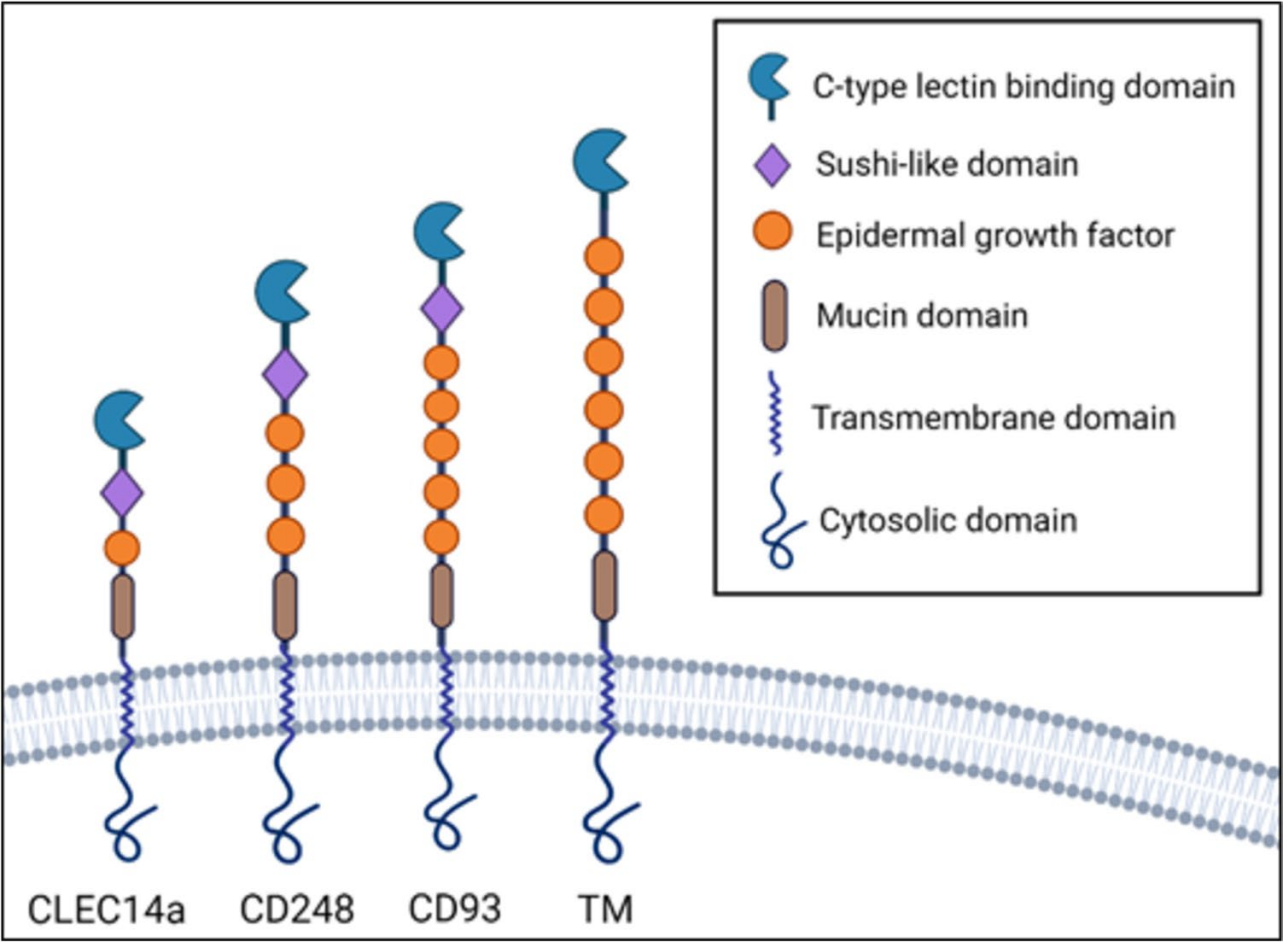
Structural representation of group XIV C-type lectin domain proteins in transmembrane state. CLEC14a, C-type lectin 14a, *TM* thrombomodulin

Group XIV subfamily of CTLD proteins are a relatively recent discovery among C-type lectins that share similar domain architecture, binding partners and expression, and function [18]. This family is composed of four members, including CD93, TM, CD248, and CLEC14a (Fig. 1). These proteins share a common architecture: an *N*-terminal signal peptide, CTLD containing conserved six cysteine residues (in addition to two non-canonical cysteines, except thrombomodulin which only has 4 conserved cysteine residues) followed by sushi domain (also known as complement control protein domain/short consensus repeat), a serine-proline-threonine rich region on the extracellular domain that allow for frequent modifications, namely glycosylation, a single pass transmembrane portion, followed by a cytoplasmic domain [19]. Each protein is distinguished by a variable number of epidermal growth factor (EGF) subunits. All of them can be membrane-bound or secreted in a soluble form, the latter of which often occurs via metalloproteases [20]. All group XIV proteins are preferentially expressed by mesoderm-derived cells such as endothelia and hematopoietic cells for TM, CD93, and CLEC14a, and mesenchymal cell-specific expression of CD248. Furthermore, shared binding partners amongst the group XIV proteins support their homology; CD248, CD93, and CLEC14a bind to endothelial extracellular matrix (ECM) protein multimerin-2 (MMRN2), although at different regions of their extracellular domains; TM and CD248 bind ECM glycoprotein fibronectin while TM and CLEC14a are known to both bind heat shock protein 70-1 (HSP70-1). [21, 22]

In normal physiology, group XIV CTLDs have key roles in maintaining homeostasis by regulating vessel formation, ECM organization, and inflammatory responses. Thrombomodulin acts as a natural anticoagulant by binding to the serine protease thrombin which renders thrombin unable to convert fibrinogen to fibrin, ultimately prohibiting formation of a reactive platelet plug [23]. CD248 is unique in its expression on mesenchymal cells, particularly cells of blood vessel wall called pericytes that are responsible for vessel integrity [24]. CD93 and CLEC14a are proteins minimally expressed on endothelia but upregulated during intense angiogenesis of embryogenesis and in other pathologic conditions including inflammation and malignancy, facilitating vessel tube formation, endothelial adhesion and migration, and orchestrating surrounding stroma [25–27]. Taken together, the group XIV CTLDs, while slightly variable in structure and binding interactions, all appear to serve important roles in vasculogenesis.

Over the past thirty years, the role of group XIV CTLDs and C-type lectins at-large in cancer progression has risen to prominence. Various CTLD families such as selectins, dectins, and related lectins such as galectins are among the most well characterized glycoproteins harboring carbohydrate binding domains implicated in facilitating a pro-tumorigenic TME and metastasis, predominantly via recruitment and adhesion of various immune and hematopoietic cells, and even tumor cells themselves [28]. Among group XIV proteins, CD248, originally identified as a human fetal fibroblast antigen, was found to be the most upregulated endothelia-related gene in CRC tissue compared to normal tissue, thus garnering the designation, tumor endothelial marker 1 [29]. CD93 and CLEC14a were similarly among the most enriched genes comprising an angiogenesis signature of over 1000 tissue samples across three carcinomas [30]. The involvement of Group XIV proteins in tumor angiogenesis is without question, yet, the molecular mechanisms and implications for tumor progression and abrogation are still in their nascent phases. The following sections will review our current understanding of each group XIV protein’s known role in tumorigenesis, discuss therapeutic endeavors, and offer potential directions for future study.

### CD93: overview

CD93 is a group XIV CTLD with five repeating EGF domains primarily expressed by endothelial cells (EC), neurons, and a mirage of myeloid cells such as macrophages, monocytes, and stem cells [18]. Previously referred to as complement component C1q receptor (C1qRp), it is now known that CD93 does not directly binding this complement protein but associates with various ECM proteins in the absence of calcium [19, 31]. In fetal mice, CD93 has been shown to be ubiquitously expressed on primitive hematopoietic cells, aorta, gonads, and mesonephros regions, with a predominant role in vascular remodeling given its specific expression on expanding branches of the aorta and neural plexuses on embryonic day 9–10. [32] Nonetheless, absence of CD93 during embryogenesis does not cause fatal consequences suggesting redundancy in its function. In adult humans and mice, CD93 expression wanes.

Thus far, facilitation of efferocytosis (removal of apoptotic cells), endothelial maturation, migration, and intercellular adhesion are proposed native functions of CD93. Rather than directly binding complement receptors, as once regarded, in vitro studies using transfected Chinese hamster ovary (CHO) cells, THP-1 macrophages, and primary human macrophage cultures, soluble CD93 has been shown to act as an opsonin to mediate macrophage recruitment and phagocytosis [33, 34]. As a cell-surface protein, CD93 appears to mediate endothelial function, namely adhesion with other ECs, migration, and interaction with the ECM, via interaction with various binding partners such as Multimerin-2 (MMRN2), *β*-dystroglycan, Cbl, and recently insulin growth factor binding protein 7 (IGFBP7) [14, 35, 36]. A series of studies from the University of Siena has elucidated much of our mechanistic understanding of CD93 interaction with MMRN2 and downstream pathways facilitating EC function. First, the importance of MMRN2 interaction with CD93 in promoting endothelial function was demonstrated by blocking CD93/MMRN2 interaction with a monoclonal antibody (mAb) against the CTLD of CD93 and resultant inhibition of human umbilical vein endothelial cell (HUVEC) adhesion, migration, and tube formation [35]. Next, elucidation of amino acid residues important for interaction between CD93 and MMRN2 via mutagenesis assays revealed proper folding of the CTLD and presence of a phenylalanine residue at position 238 to be crucial in binding MMRN2 and carrying out angiogenic roles [37]. Subsequently, the Siena group showed that CD93 interaction with β-dystroglycan, an ECM protein upregulated on activated ECs, is also important for endothelial activity via phosphorylation of CD93 extracellular domain, which in turn stimulates phosphorylation of downstream effectors such as Cbl [36]. Most recently, they demonstrated that CD93 preserves endothelial junctions via VE-cadherin interaction and suppression of phosphorylation via Rho signaling, thereby mitigating vessel permeability [38]. Recombinant forms of soluble CD93 comprised of only EGF repeats and the mucin domain of its CTLD have been shown to retain pro-angiogenic stimulation on ECs in vivo with perhaps more potent angiostimulation via the EGF domains [39]. Together, this data clearly defines a role for CD93 in mediating angiogenic processes and requires specific ligands and environments, such as malignancy, to execute its function.

### CD93: role in malignancy

While CD93 is preferentially expressed on ECs, they are at a minimal or absent level in normal human and mouse adult tissue. Recent studies have shown CD93 to be intensely upregulated on tumor-associated endothelium, supported by its status as one of the top 20 genes associated with human primary tumor angiogenesis signature surveying head and neck, breast, kidney, and brain tumors [30]. In subsequent studies, CD93 has been shown to be a downstream effector of VEGF, a potent stimulator for tumor-derived angiogenesis, and its expression significantly downregulated upon VEGF inhibition [14]. From single cell gene expression to surface protein expression, CD93 expression is by-far predominantly upregulated on solid organ malignancies and, to a lesser extent, some hematologic malignancies. In a human pan-cancer analysis of transcriptomic and mutational data from several online databases, CD93 was found to be upregulated in most cancers such as cholangiocarcinoma, ovarian, pancreatic, gastric, melanoma, and kidney but down regulated in lung adenocarcinoma and bladder urothelial cancer [40]. Prognostically, higher CD93 expression levels were correlated with worse oncologic outcomes in kidney renal papillary carcinoma, glioma, ovarian cancer, and uveal melanoma. In this same CD93-specific screening investigation, increased CD93 expression correlated with higher number of infiltrating T cells in some cancers but also higher immunosuppressive macrophage presence [40]. Immune-related genes known to promote cancer growth, tumor angiogenesis, and tumor metastasis were also positively correlated with CD93 upregulation. CD93 expression and upregulation on tumor endothelium has also been confirmed with immunostaining. Langenkamp et al. demonstrated that among low to high grade gliomas, higher grade gliomas had tumor vasculature with higher degree of CD93 positivity compared to lower grade gliomas [41]. In another study of human gliomas, Ma et al*.* showed CD93 expression was positively correlated with infiltration of immunosuppressive immune cells such as immunosuppressive macrophages, T regulatory cells, and myeloid derived suppressor cells (MDSCs) [42]. Similar cell-surface staining has been shown in carcinomas of the kidney, colon, and pancreas, supporting the notion that CD93 is upregulated on blood vessels supporting several types of tumors. [14]

In vitro and in vivo preclinical models exploring the roles of CD93 in angiogenesis, tumor progression and metastases build upon the foundation of our current mechanistic understanding of CD93. In in vitro studies of human dermal microvascular endovascular cells (HDMECs) treated with VEGF, CD93 knockdown cells failed to engage in cell adhesion and form tubes compared to untreated cells but rescued with lentivirus transfection of wild type (WT) CD93 with native cytoplasmic region [38]. Further, CD93 expressing ECs became disorganized and lost adhesion properties upon CD93 knockdown. In preclinical in vivo models, Langenkamp et al*.* demonstrated CD93 KO (KO) mice with orthotopically implanted mouse gliomas experienced longer survival and slower tumor growth compared to control mice [41]. Further, in subcutaneous fibrosarcoma model, CD93 KO mice had reduced tumor size. In histologic and immunofluorescent analyses, gliomas in CD93 KO mice were infiltrated by endothelia with abnormally polarized lumens and increased vascular permeability compared to controls, suggesting a high degree of vessel dysfunction in the absence of CD93.

Ligand interaction with CD93 appears to mediate its role in physiologic endothelial activity as well as tumor angiogenesis. Analysis of The Cancer Genome Atlas and endothelial specific online database (EndoDB) demonstrate that IGFBP7, a secreted ECM protein implicated in a variety of homeostatic processes, is upregulated on tumor endothelium [43]. Our lab has recently demonstrated specificity of IGFBP7 binding to CD93 [14]. Out of a library of over 6000 surveyed cell surface and soluble proteins, not including MMRN2, IGFBP7 was the only positive binding partner with CD93. The knockdown of IGFBP7 in HUVECs prevented tube formation compared to WT controls. Further, the importance of CD93 binding to IGFBP7 for angiogenesis was demonstrated by knockdown of CD93 in HUVECs and the absence of tube formation and EC migration with the addition of IGFBP7 protein but restored function with the addition of IGFBP7 in WT HUVECs. Likewise, the role of CD93-IGFBP7 interaction in tumor angiogenesis seems to be important. Blockade of the interaction with anti-IGFBP7 antibody similarly reduced tumor growth while promoting tumor vessel maturation, as evidenced by increased pericyte coverage and reduction in integrin *β*1/CD29 activation, a marker of EC destabilization and vascular leakiness. Similarly, Xu et al*.* implanted murine melanoma tumors with and without transfected IGFBP7 and found that IGFBP7 tumors had significantly more intratumoral vessels on immunostaining [44]. Furthermore, expression of IGFBP7 mutants that lose CD93 binding in tumors did not increase vessel densities as seen in tumors expressing WT IGFBP7, implicating a role of IGFBP7 in upregulating tumor angiogenesis via CD93.

### CD93: therapeutic approaches

**Fig. 2:**
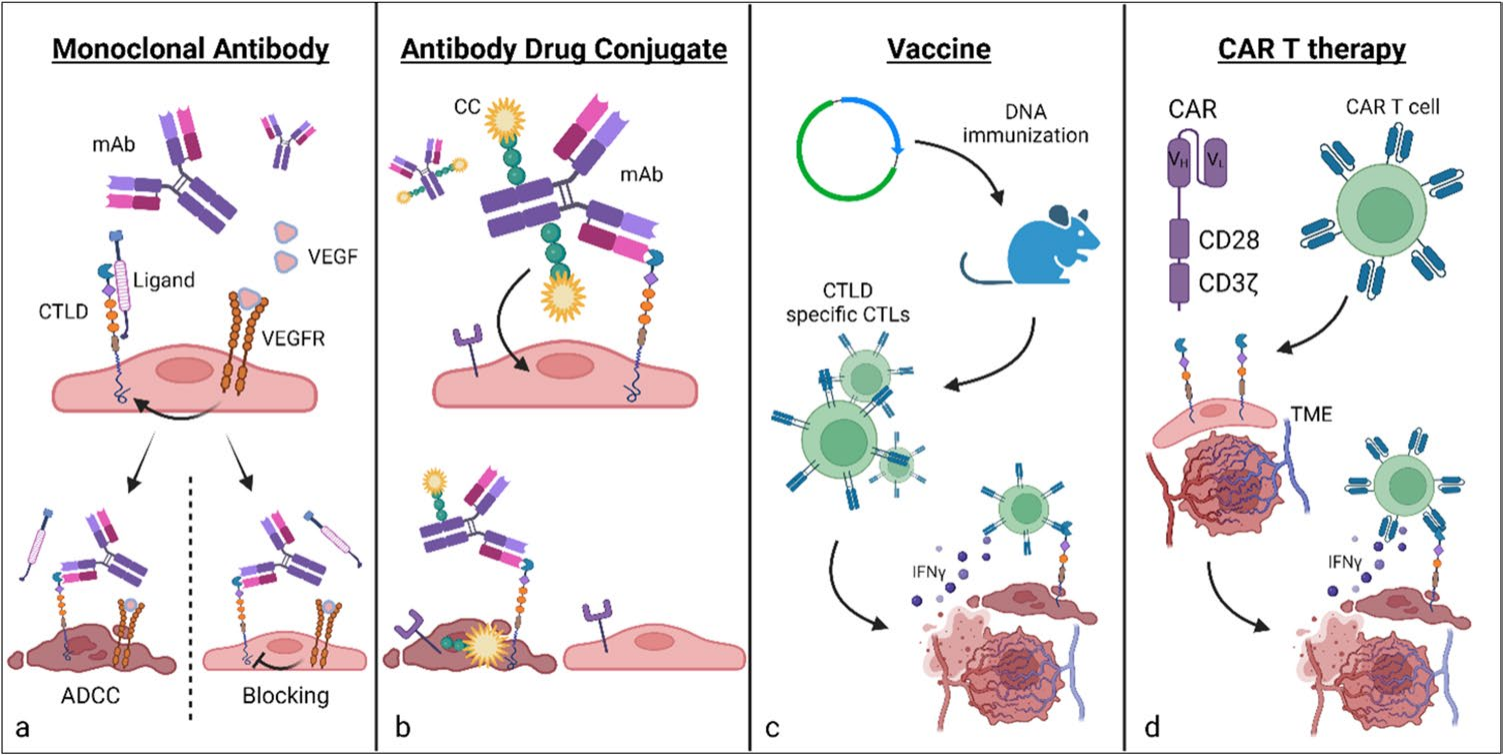
Examples of anti-tumor therapeutic approaches targeting Group XIV CTLDs. **a** Monoclonal antibody (mAb) specifically targeting CTLD with either cellular depleting effects via antibody-dependent cell mediated cytotoxicity (ADCC) or blocking function inhibiting downstream signaling **b** Antibody drug conjugate (ADC) targeting CTLD to induce receptor mediated endocytosis of cytotoxic conjugate (CC) in form of DNA intercalators or inhibitors of cell division resulting in cell death **c** Anti-cancer vaccination with immunogen such as fused DNA construct of CTLD and tetanus toxoid stimulating CTLD specific cytotoxic *T* lymphocytes (CTLs) targeting malignant CTLD-expressing cells **d** Chimeric antigen receptor *T* (CAR *T*) cell therapy engineered with high specificity antibody binding fragments (V_H_ & V_L_) targeting CTLD and intracellular *T* cell stimulating motifs to activate cytotoxicity via cytokines (interferon gamma). *VEGF/R*, vascular endothelial growth factor/receptor

Recent work detailing blockade with a CD93 mAb has led to interesting and promising results toward tumor vessel normalization rather than depletion as seen in antibody-directed binding to other group XIV CTLDs such as CD248 and CLEC14a as will be discussed in subsequent sections (Fig. 2a). CD93 signaling inhibition with anti-CD93 mAb led to tumor vessel normalization rather than depletion as evidenced by maturation of blood vessels with increased pericyte coverage via upregulated immunofluorescent staining of alpha-smooth muscle actin (*α*SMA) and neural/glial antigen 2 (NG2) coverage of vessel structures [14]. Additionally, blockade of CD93 resulted in improved perfusion of subcutaneously implanted pancreatic and melanoma tumors, suggested by decrease in intratumoral hypoxia with improved tomato lectin staining of intratumoral vessels and hypoxic inducible factor 1 alpha (HIF1α) expression—this is in direct opposition to results reported in CD93 KO gliomas where CD93 knockdown mice exhibited worse perfusion than WT counterparts [41]. The vessel maturation induced by CD93 blockade resulted in exciting downstream therapeutic benefits—subcutaneous melanoma and pancreatic tumors had decreased growth, improved efficacy of chemotherapy delivery, and increased influx in anti-tumor immune cells such as CD8 + *T* cells and natural killer cells synergizing the effect of immunotherapy. However, the effects of CD93 blockade on orthotopic tumors, known to display tumor microenvironments more similar to human cancers, is a subject of ongoing investigation. Nonetheless, these promising preclinical results have led to initiation of a phase one clinical trial (Study DC-6001-101) (Table 1). [45]

**Table 1:** Summary of group XIV CTLDs’ characteristics

Protein	# of EGF domains	Cell expression	Expression level, healthy adult	Normal physiologic role	Malignant expression. pattern	Role in tumor progression/angiogenesis	Preclinical therapeutics	Clinical trial status
CD93	5	EC, neurons, monocytes, platelets	low	EC adhesion, migration, vessel tube formation; stabilize EC junctions	Upregulated	Stimulated by VEGF; interacts with MMRN2, IGFBP7, β-dystroglycan to promote vessel growth; vessel polarization	mAb	Phase I (DCBY02-101, advanced or metastatic solid organ malignancies)
CD248 (endosialin)	3	Fibroblasts, pericytes, MSCs	low	Stromal cell adhesion, migration; Remodeling of ECM	Upregulated	Interacts with MMRN2 to remodel ECM, promote vascular permeability	mAb, ADC, vaccine, CAR T therapy	Phase I, II (MORAb-004, solid organ malignancies, metastatic CRC/melanoma/sarcoma)
CLEC14a	1	EC	low	EC adhesion, migration, vessel tube formation; regualete	Upregulated	Flow regulated; binds MMRN2 to promote angiogenesis	mAb, CAR T therapy, nanoparticle delivery of CLEC14-targeted siRNA	–
CD141 (TM)	6	EC, vascular SMC, epithelial cells, keratinocytes	moderate/high	Binds thrombin—> activation of protein C; activates TAFI—> promote coagulation; antiinflammation, cytoprotection	Cell surface: downregulated; soluble: upregulated	Reduction leads to increased metastases; regulates production of EMT proteins; inhibits TAFI activation limiting plasmin and MMP9 production; facilitate cell adhesion and angiogenesis; inhibits cell proliferation	CD141 + DC vaccine, exogenous TM targeting dissolution of cancer-associated NETs	–

*TM* thromobmodulin, *EC* endothelial cell, *MSC* mesenchymal stem cell; *SMC* smooth muscle cell, *ECM* extracellular matrix, *VEGF* vascular endothelial growth factor, *MMRN2* Multimerin 2, *IGFBP7* insulin growth factor binding protein 7, *mAb* monoclonal antibody, *ADC* antibody drug conjugate, *CAR T* chimeric antigen receptor *T* cell therapy, *siRNA* silencing RNA, *CRC* colorectal cancer, *EMT* epithelial to mesenchymal transition, *TAFI* thrombin activating fibrinolysis inhibitor, *MMP9*, matrix metalloprotease 9, *DC* dendritic cell, *NETs* neutrophil extracellular traps

CD93 has been demonstrated to play a significant role in vessel formation, both by normal and pathologic processes. Its upregulation in a plethora of solid organ tumors, and promising anti-tumor effects when disrupted, especially given its distinct vascular normalization effects, give credence to further investigation into how CD93 acts as an intercellular adhesion molecule and promoter of EC formation. Further, the expression of CD93 on EC of the lung and liver gives impetus to investigate the normal physiologic function of CD93 in these organs and how this function is dysregulated, if at all, in metastatic dissemination.

### CD248: overview

CD248, also named endosialin due to its high degree of glycosylation, shares the common structure with its fellow group XIV CTLDs with the exception of containing three EGF repeat domains [26]. Like CD93, CD248 is absent (brain, stomach, skin, ovary) or minimally expressed (small intestine, uterus, kidney) on adult normal tissue but pronounced during fetal development, yet deficiencies in CD248 expression during this stage seem to be produce no postnatal effects [46]. CD248 is unique from its group XIV kin in that it is not expressed on ECs, contrary to its previous name tumor endothelial marker 1, but only mesenchymal cells including stromal fibroblasts, smooth muscle cells, pericytes, mesenchymal stem cells, and naïve T cells [47, 48]. Its role during embryogenesis involves vascular remodeling and angiogenesis. In vitro studies suggest CD248 remodels developing vasculature via vessel pruning and branch regression [49]. Further, the silencing of CD248 in cultured human fibroblasts leads to reduced capacity to migrate and proliferate. CD248 has also been shown to have a role in promoting inflammation—in autoimmune diseases, CD248 is known to be upregulated on fibroblasts and pericytes of synovial tissue and mesenchymal cells of the skin as KO of CD248 cytoplasmic domain showed significant reductions in inflammatory cytokines, synovitis, and arthritis in murine models. [19, 50]

Mechanistically, CD248 is proposed to exist within a complex interplay involving various ECM proteins and signaling pathways mediating stromal cell migration, activation, and proliferation. CD248 has been shown to bind to ECM proteins collagen I, collagen IV, and fibronectin [51]. In a series of elegant in vitro studies, Tomkowicz et al*.* demonstrated that CD248 expression level correlated with fibronectin expression, and that CD248 expression facilitated adhesion of cells to fibronectin, resulting in unique, web-like morphology and enhanced migratory capacity of CD248 expressing CHO cells. Furthermore, production and secretion of matrix metalloprotease-9 (MMP-9), a known enzymatic protein responsible for basement membrane remodeling, was found to be upregulated in the supernatant of CHO expressing CD248 cells, which the authors hypothesized could contribute to the enhanced migratory phenotype of CD248 CHO cells in vitro. Importantly, the authors also developed a humanized IgG mAb to CD248 (MORAb-004, commercially ontuxizumab) that blocked CD248 binding to its ECM ligands. MORAb-004, as discussed in subsequent sections, demonstrated the validity of CD248 targeting in preclinical tumor models, and has advanced to clinical trials.

The cytoplasmic domain of CD248 seems to facilitate angiogenesis-independent stromal activity. Maia et al*.* demonstrated that the presence of CD248 cytoplasmic domain suppresses expression of tumor suppressor transgelin (SM22a), a repressor of transforming growth factor-beta (TGF-*β*) and MMP-9 expression, thus the absence of the cytoplasmic domain upregulated SM22a and decreased MMP-9 activity [22]. Furthermore, in a transwell migration assay, WT fibroblasts expressing CD248 migrated much further than CD248 cytoplasmic domain null counterparts in a platelet-derived growth factor-BB (PDGF-BB) dependent manner, suggesting migration is facilitated by the cytoplasmic domain. Lastly, tumor cell viability may be affected by loss of CD248 cytoplasmic domain as coculture of WT and cytoplasmic domain-absent CD248 with T241 fibrosarcoma cells resulted in reduced tumor cell viability after 48 h. In sum, CD248 uses a variety of mechanisms to facilitate stromal remodeling, many of which are dysregulated in pathologic conditions.

Interestingly, CD248 has also been shown to be expressed on a specific subset of human naïve CD8 T cells in the thymus and peripheral blood. The definitive role of CD248 on *T* cells has yet to be elucidated, however Hardie et al*.* has begun to explore the anti-proliferative function of endosialin in vitro [48]. They speculate that the decrease in CD248 expression in extrafollicular zones of secondary lymphoid structures retains CD8 + *T* cells in antigen recognition areas, promoting a quiescent *T* cell state. However, the anti-proliferative role of CD248 on CD8 + *T* cells has not been demonstrated given the lack of expression on murine *T* cells nor is it known if tumor associated CD8 + *T* cells express CD248 differentially than other effector *T* cells.

### CD248: role in malignancy

CD248 was originally deemed TEM1 given its status as the highest upregulated gene in a seminal serial analysis of gene expression (SAGE) of endothelial genes found in malignant CRC samples compared to normal tissue [29]. This understanding has since been corrected as studies have demonstrated CD248 to not be expressed by endothelia but by cells supporting the tumor environment, namely mesenchymal cells such as pericytes and activated fibroblasts, and tumor cells themselves, mostly of the mesenchymal type such as sarcomas but also of epithelial origin such as CRC [52–54]. In a seminal study by Christian et al., immunohistochemical (IHC) staining of human CRC tissue revealed colocalization of alpha smooth muscle actin (αSMA) and podoplanin, conventional markers of cancer-associated fibroblasts, and CD248 [55]. In the same study, more aggressive human melanoma samples exhibited stronger CD248 staining of the myofibroblasts compared to less advanced tumors suggesting that CD248 upregulation is correlated with tumor biology. Similarly, Hong et al*.* found that more aggressive human lung adenocarcinomas express higher levels of CD248 along with ligands OPN and SERPINE1 [56]. Further, higher expression of these genes was correlated with worse overall survival. In histologic analysis, larger lung tumors were found to have higher counts of pericyte positive CD248, OPN, and SERPINE1 compared to smaller tumors. In a case series of brain tumors, Brady et al*.* demonstrated that all examined human brain tumors expressed CD248, however, there was a gradient of highest to lowest expression of CD248 on tumor associated vessels in most advanced stage to lower stages, respectively. [57] In contrast, O’Shagnnessy et al*.* conducted single cell RNA sequencing and IHC analysis of cohort of sarcomas at their institution and found that higher CD248 expression determined by IHC analysis but not sequencing data was correlated with improved overall survival [58]. Explanation as to why prognosis for higher expressing sarcomas is improved compared to other cancers perhaps lies in a differential in the tumor cell vs tumor stroma expression. Kondo et al. analyzed 18 human samples of osteosarcoma (OS) via IHC staining for CD248 and found that metastatic OS had significantly higher expression of CD248 [15].

In mouse models CD248 KO has been shown to have tumor abrogating effects via vascular depletion. In a study of orthotopic lung tumor implantation, mice with CD248 KO demonstrated smaller tumors compared with control [56]. This gross difference in size was explained by depletion of tumor associated vasculature, increased dysfunctional vessels, and increased intratumoral hypoxia via loss of CD248 positive pericyte coverage and downregulation of WNT signaling pathway. In Lewis lung carcinoma (LLC) tumors implanted in the colon and orthotopic glioblastomas, CD248 KO tumors demonstrated significantly more small vessels (< 50um) within the tumor compared to controls [46, 59]. Interestingly, preclinical studies suggest a site-specific response to CD248 in the progression of primary tumors. Nanda et al*.* compared tumor growth in subcutaneous vs abdominally implanted tumors and found that there was no difference between growth curves and local invasion in subcutaneously implanted lung carcinomas compared to stark decrease in liver and colon implanted tumors in KO vs WT CD248 mice [46]. IHC analysis revealed significantly higher density of smaller blood vessels in CD248 KO tumors.

In preclinical metastatic models, CD248 KO mice similarly shows favorable results. Nanda et al*.* implanted human CRC xenografts into colonic wall of WT and CD248 KO mice [46]. KO mice were observed to have decreased peritoneal carcinomatosis and absence of liver metastases compared to controls. Viski et al. orthotopically injected breast cancer and implanted subcutaneous LLC cell lines in KO versus WT mice and observed no difference in primary tumor size but a significant reduction in lung metastases, a result shown not to be dependent on anti-tumor immunity [60]. Interestingly, in their histologic and functional analysis of primary tumors, there was no difference in the microvessel density (MVD), hypoxia, degree of *α*SMA staining on pericytes as reported by previous studies. Further, in a hematogenous lung metastasis model via tail vein injection, they observed no difference in lung tumor burden concluding that CD248 does not affect the seeding of disseminated tumor cells in distant organs. Enhanced intravasation of tumor cells across endothelium into the vessel lumen via stromal CD248 expression was demonstrated to be dependent on direct contact with pericytes expressing CD248. Finally, in human breast carcinomas samples, Viski et al*.* also showed that primary tumors with higher stromal CD248 expression correlated with decreased recurrence free survival; mRNA sequencing analysis revealed a positive relationship between CD248 levels in the primary tumor and increased incidence of metastases. The authors concluded that rather than intrinsically altering tumor cell metastatic potential, CD248 expressing pericytes facilitate metastasis by mediating transmigration through basement membrane and into the endothelium. Supporting this, Kondo et al*.* demonstrated that human OS cell lines incubated with humanized anti-CD248 mAb, MORAb-004, inhibited cell migration in the presence of fibronectin but did not demonstrate any cytostatic effects in vivo*.* [15]

### CD248: therapeutic approaches

Specific targeting of tumor associated CD248 has naturally given way considering its apparent tumor specificity. Since its inception in 2007, humanized mAb to CD248, MORAb-004, has been the predominant prototypical therapeutic antibody used in the preclinical and clinical setting. Using human CD248 knock-in mice, Rybinski et al*.* demonstrated that MORAb-004 treatment significantly reduced tumor burden in both subcutaneous primary and hematogenous lung metastasis models [61]. Furthermore, consistent with CD248 KO data, tumors treated with MORAb-004 exhibited an increase in small, nonfunctional blood vessels that lost normal polarization. It was also found that with antibody treatment, cell-surface CD248 was internalized in the cell, resulting in reduced levels of cell surface CD248. Interestingly, αSMA expression was also significantly reduced after antibody blockade, a potential corollary to the heightened degree of tumor vessel dysfunctional after CD248 mAb blockade.

The promising results garnered in the pre-clinical setting has led to MORAb-004 entering early phase clinical trials although thus far has demonstrated limited therapeutic advantage. A phase I clinical trial in 36 patients with refractory solid organ tumors such as CRC, hepatocellular carcinoma (HCC), and pancreatic adenocarcinoma (PDAC) were treated with once weekly dose of MORAb-004 [62]. A third of patients had stable disease for slightly over three months, including four who had minor favorable anti-tumor radiographic effects. Two randomized phase II trials in chemo-refractory metastatic CRC and metastatic melanoma patients followed, both of which demonstrated no difference in progression-free survival, overall survival, or overall response rate compared to placebo, and stable disease in 40% [63, 64]. In part 2 of a phase I randomized clinical trial in metastatic sarcomas, MORAb-004 was added to combination gemcitabine and docetaxel; there was no difference in progression-free or overall survival compared to placebo. [65]

In addition to specific antibody binding blockade, various novel immunotherapies including antibody drug conjugates (ADCs), cancer vaccines, and chimeric antigen receptor *T* (CAR *T*) cell therapy directed against CD248 have been explored. One group developed a CD248-specific ADC by conjugating monomethyl auristatin E (MMAE), a known potent cytotoxic anti-mitotic agent, to mAb against CD248 (Fig. 2b) [52]. Sustained antitumor response was observed in survival experiments using human neuroblastoma and Ewing sarcoma xenografts in nude mice, with complete reductions in tumor volumes and prolonged median survival of nearly 100 days compared to the admix control (anti-CD248 + MMAE separate) with median overall survival of 40–50 days in both cell lines. Cancer vaccination is a hot area of investigation for immunotherapy. Facciponte et al*.* fused full length CD248 antigen with adjuvant tetanus toxin to create a TEM-1 specific vaccine with promising effect in both prophylactic and therapeutic setting (Fig. 2c) [66]. Subcutaneously implanted murine lung and colorectal tumors were significantly smaller with compromised perfusion and microvascular density and increased tumor cell apoptosis compared to control and TEM1 alone constructs, which the authors found to be driven by cytotoxic *T* cell driven immune response to tumor vasculature associated structures. Further, they found that TEM1-TT inoculation induces cross-priming of other tumor-specific antigens in murine colorectal and lung carcinoma models, compounding active cellular immune response against tumor cells. Novel advances in CAR *T* cell therapy have led to the development of bispecific “*T* cell engagers” also referred to as (BiTEs) that possess high affinity antibody fragments toward CD3 and tumor antigen. Recently, Fierle et al. have engineered a trivalent BiTE against CD248, demonstrating enhanced activation of CD248 specific cytotoxic *T* cell activity in vitro and in subcutaneous xenograft murine models [67]. Taken together, targeted immunotherapy approaches against CD248 appear promising and specific.

While the clinical trial data supporting the clinical efficacy of CD248-specific mAb therapy has yet to be produced, there is little doubt of the specificity and potential impact targeting CD248 has on treating solid organ malignancies. The expression on CD248 on activated fibroblasts and other mesenchymal cells critical to supporting tumor vasculature opens an exciting door to potentially whole-scale alter the TME by targeting a tumor’s stromal component, a known yet challenging barrier to maximizing the efficacy of immunotherapy writ-large.

### Thrombomodulin: an overview

Thrombomodulin (CD141, and BDCA3; TM) is a XIV CTLD member unique in structure, expression pattern, and function. Like CD93, CD248, and CLEC14A, TM has a classic CTLD binding domain with six variable repeat EGF domains, however, it lacks a sushi domain which is conserved in the other members of group XIV [19]. In the transmembrane form, TM contains a single pass domain; soluble TM, a result of proteolytic cleavage from the cell surface, circulates in the blood, urine, and various other biofluids. TM appears to be vital to embryonic development as mice lacking TM, which are predominantly expressed by trophoblasts of the placenta, undergo intrauterine death due to trophoblast apoptosis and a complete loss of diploid trophoblast proliferation. [68, 69]

In healthy human adult tissue, TM is expressed ubiquitously on a wide array of cells of the vascular endothelia, lymphatic endothelia, mesothelia, astrocytes, keratinocytes, osteoblasts, chondrocytes, alveolar epithelial cells, and various hematopoietic cells [70]. TM facilitates several homeostatic processes via high affinity ligand binding to its CTLD and EGF domains. Currently, it is believed that TM does not possess any intrinsic enzymatic properties nor directly activates intrinsic signaling pathways, rather, it serves as a critical facilitator of several physiologic processes by altering the specificity and potency of downstream enzymatic reactions.

A prime example of this facilitating capacity is TM’s role in anticoagulation. Membrane-bound TM binds thrombin to block its interaction with procoagulants, such as fibrinogen and protease-activated receptor (PAR)-1; as a result, this process substantially increases the specificity of thrombin for Protein C, its subsequent activation, and enhances inactivation of downstream pro-coagulant factors [23, 68, 71]. Other processes whereby TM serves a mediator function include regulation of cell adhesion and inflammation through a variety of mechanisms, some of which are independent of thrombin binding [70, 72]. Like CD248, TM promotes cell adhesion via binding of fibronectin to its CTLD, a thrombin-independent process. Thrombin activatable fibrinolysis inhibitor (TAFI), an enzyme activated via the TM-thrombin complex, plays an anti-inflammatory role by inactivating pro-inflammatory cytokines and inhibiting inflammatory complement proteins C3a and C5a [73]. Further, TM indirectly enhances cytoprotective mechanisms via generation of activated protein C (APC) and inhibition of NF-kB signaling and nuclear translocation [72, 74].

Regarding angiogenesis, studies have shown that different domains of TM mediate angiogenesis in contrasting ways. The EGF domains and serine/threonine-rich area appears to promote angiogenesis via increasing EC proliferation and migration while the CTLD of TM seems to either facilitate vasculogenesis via promotion of cell–cell adhesion or inhibiting angiogenesis by interfering with endothelial EGF receptor interaction with Lewis Y antigen [75–77]. Physiologic context likely dictates which angiogenic function TM facilitates. In a pro-angiogenic setting, like CD93, HUVEC-expressed TM is upregulated after VEGF stimulation in a dose-dependent fashion [77]. Furthermore, in vitro TM knockdown reduced VEGF mediated HUVEC adhesion and migration via fibronectin, akin to CD248-fibronectin interaction. Additionally, TM has been proposed to interact with novel G-protein coupled receptor 15 (GPCR15) expressed on activated ECs to generate nitric oxide (NO) and promote angiogenesis while enhancing cytoprotective mechanisms [78]. APC formation facilitated by TM-thrombin binding binds to its receptor endothelial protein C receptor (EPCR) to activate downstream signaling promoting tube formation and EC protection [79]. The CTLD like domain, while serving as mediator of cell–cell adhesion, has also been reported to inhibit angiogenesis. In a series of recombinant TM assays utilizing different TMs with varying extracellular domains, Kuo et al*.* observed that TM containing only the CLTD inhibited HUVEC angiogenic phosphorylation-driven signaling and EGF receptor interaction with Lewis Y antigen [75]. This specific function was extended to decreased angiogenesis in in vivo Matrigel plug assays and further demonstrated in reductions in LLC xenograft MVD and gross tumor size upon treatment with CTLD-only TM.

### Thrombomodulin: role in malignancy

Like the other group XIV CTLDs, TM’s various physiologic roles have important implications in regulating cancer progression. Unlike CD93 and CLEC14a but like CD248, expression of TM on tumor cells themselves seems to carry more relevancy to cancer biology than angiogenesis. Importantly, unlike its group XIV relatives, TM expression appears to have a protective or anti-tumorigenic effect in several solid organ malignancies—higher expression of TM in a host of cancers such as lung adenocarcinoma, invasive breast carcinoma, oral squamous cell carcinoma (SCC) and esophageal SCC, HCC, CRC, and PDAC has been correlated with less aggressive tumors and improved patient survival [80–83]. Soluble TM also has been shown to have role in cancer progression, but in contrast to membrane bound TM, soluble forms seem to be upregulated in advanced cancer likely due to increased shedding from cancer cells themselves [84, 85]. TM involvement in a wide array of physiologic processes, in both membrane bound and soluble forms, results in diverse and complex consequences in malignancy.

The regulation of tumor progression by membrane-bound TM acts predominantly by decreasing tumor cell proliferation and mitigating various steps of the metastatic cascade, however, conflicting data in opposition to TM’s anti-tumor properties do exist. Zhang et al. demonstrated that TM expression was inversely related with degree of cell proliferation in subcloned human melanoma tumor cell lines [86]. In the same report, overexpression of TM in murine melanoma cell lines resulted in reduced tumor cell division in an APC independent fashion compared to WT cells expressing normal levels of TM. Knockdown of TM in human bladder cancer cell lines resulted in an increase in primary tumor size [83]. In contrast to these findings, Horowitz et al*.*, showed that although mutated TM without ability to bind thrombin significantly increased hematogenous metastases in murine melanoma, no difference in primary tumor growth between thrombin-binding mutant, lectin-domain mutant, and WT was observed [71]. Considered collectively, TM appears to be distinct from other Group XIV CTLD members in that it has a predominantly tumor-limiting role, the mechanisms of which warrants further exploration.

Regarding TM involvement in metastatic dissemination, Horowitz et al. employed in vivo murine melanoma models comparing transgenic TM mutants (thrombin binding domain, lectin-domain mutated mice) to demonstrate TM’s anticoagulative properties via thrombin binding are essential to limiting metastatic seeding and tumor cell survival in distant organs [71]. Further, the knockdown of TM has been shown to result in an increase in proteins involved in epithelial to mesenchymal transition (EMT) such as vimentin, Snail, and ZEB1. TM activates TAFI which inhibits plasmin and limits the breakdown of ECM, thus preventing metastatic extravasation. Furthermore, by forming a complex with thrombin, TM inactivates platelets which are known to protect disseminated tumor cells via platelet aggregation. In a recent study by Kawamoto et al., the authors proposed TM contributes to limiting metastases by binding to ECM protein fibronectin, blocking adhesive integrins expressed by breast carcinoma cells from binding fibronectin and initiating tumor cell migration [82]. This mechanism adds novel depth to the previously demonstrated understanding that TM binding fibronectin promotes tumor cell adhesion, migration, and angiogenesis [77]. Finally, the cytoplasmic tail of TM has been implicated in interacting with intracellular ezrin, which can interact with CD44 receptor and facilitate tumor cell migration [87]. TM appears to augment the metastatic cascade through anti-coagulation, cell adhesion, and regulation of EMT proteins.

Taken together, TM predominantly executes an anti-tumor function by decreasing cell proliferation and antagonizing tumor metastasis. TM involvement in downstream pathways relevant to cancer can be thrombin dependent or independent. Just as TM balances pro- and anti-coagulative functions, so too does it seem to execute both tumorigenic or tumor suppressive roles dependent on the proximal stimulus.

### Thrombomodulin: therapeutic approaches

Considering TM’s breadth of expression and importance to several homeostatic processes, few anti-cancer TM-based approaches have been documented. However, growing interest in modulating anti-tumor immunity and limiting metastatic potential via by stimulation of TM-expressing dendritic cells (CD141 + DCs) and targeting dissolution of neutrophil extracellular traps (NETs), respectively, have been explored.

CD141 + DCs are a specialized subset of DCs that can regulate immune responses via TM changing the properties of the expressing dendritic cell. CD141 + DCs display high functional plasticity and are capable of simultaneously evoking *T* cell subtypes Th1 and Th2 responses. CD141 + DCs are essential to priming naïve CD8 + *T* cell responses and excel in cross presentation of extracellular antigens in comparison to their other antigen presenting cells. CD141 + DCs murine homolog is CD103 + DCs, and murine studies have displayed an active role of CD103 + DCs/CD141 + DCs in antitumor immunity. [88, 89]

To avoid adverse side effects caused by other motifs of cancer treatment, cancer vaccines have recently been developed. Cancer vaccines attempt to activate the immune system to respond to malignancy via various mechanisms including activation of cytotoxic and cytokine producing T cells. Various sets of immune cells are utilized in cancer vaccines including dendritic cells. Because CD141 + DCs superior antigen presenting abilities Cho et. al selected CD141 + DCs in development of their DC vaccine (CellgramDC-WT1) [90]. In vitro*,* the CellgramDC-WT1 (CDW) vaccine doubled the amount of IL-12 and significantly upregulated IFN-*γ* which are both critical inducers of pro-inflammatory CD4 + Helper *T* cells Type 1 (Th1). In vitro*,* the CDW vaccine directly increased cytotoxic *T* cell count. WT1 is an antigen commonly expressed on solid tumors and has previously been the target of immunotherapy. Cho et al*.* also determined the CDW vaccine induced cytotoxic *T* cells in a Wilms’ tumor1model in a concentration dependent manner. DCs play a critical role in immunity, and because DC don’t directly kill malignant cells DC vaccines have no adverse side effects on normal cells. Because of CD141 + DCs superior cross antigen presenting, stimulation of IL-12, IFN-*γ*, and Th1 cells make CD141 + DCs a promising target for development of DC cancer vaccines.

NETs are by-products of neutrophil activation containing protein coated nucleic acids that normally serve as “traps” for various exogenous pathogens. In cancer, NETs have been shown to serve various roles in facilitating metastases namely by acting as a chemoattractant of cancer cells to distant organs and facilitator of intravascular tumor cell adhesion [91]. Furthermore, NETs have been shown to produce damage-induced protein High Mobility Group Box 1 (HMGB1) known to drive EMT [92]. One group demonstrated that administration of exogenous TM attenuated invasiveness of PDAC cells by decreasing production of EMT-associated proteins [93]. Further, liver metastases in murine model of PDAC were ameliorated with TM treatment by blocking formation of NETs and degradation of HMGB1. This study suggests novel use of TM as an anti-cancer therapeutic having a substantial impact on a newly identified facet of solid tumor metastatic progression.

While much focus has been devoted to the (anti)coagulative properties of TM, emerging evidence suggests it has a plethora of additional functions that facilitate angiogenesis and regulation of homeostatic and host defense mechanisms. Current evidence suggests TM limits tumor progression in most malignancies, however, the definitive relationship between TM and cancer is not fully known. As a result, therapeutic strategies targeting TM are nascent yet promising.

### CLEC14a: an overview

C-type lectin family 14 member A (CLEC14a) shares the structural features group XIV proteins except it possesses a single variable EGF domain [94]. CLEC14a joins a multitude of other CLECs spread across various CTLD subfamilies such as CLEC1 part of group I and CLEC-2, CLEC-12B, and CLEC-9A members of group V [95]. Like, CD248 and CD93, it is highly expressed on fetal tissue during embryogenesis but is absent to minimal in tissue of healthy adults [96]. Interestingly, like CD93, in zebrafish and mouse embryogenesis studies, knockdown of CLEC14a at 24 h postfertilization leads to delays in vasculogenesis while KO mice demonstrate increased MVD in the brain and retinae, yet ultimately fetuses remain viable.

CLEC14a, like CD93, is primarily expressed by ECs [18]. According to the Human Protein Atlas, CLEC14a mRNA expression in healthy adult tissue is limited to endothelial tissue, adipocytes, and fibroblasts of virtually every organ. Furthermore, in a meta-analysis of nearly 5400 tissue samples from over 130 studies, Robinson et al*.* found that baseline gene expression of CLEC14a was very low overall but most highly expressed in skeletal muscle and inversely proportional to degree of shear stress in blood vessels (measured by TIE1 expression) [97]. In vitro and murine models have demonstrated a central role of CLEC14a in endothelial migration, adhesion, and tube formation. Rho et al*.* and Zhuang et al*.* demonstrated that the CTLD domain of CLEC14a was essential for endothelial adhesion, migration, and filopodia formation of GFP-tagged CLEC14a HUVECs in vitro. [25, 98] In a wound healing assay, knockdown of CLEC14a with siRNA resulted in reduced the migratory distance of silenced cells compared to WT HUVECs [25]. Similarly, Ki et al*.* mutated CTLD of CLEC14a and compared migration with WT HUVECs and found that the CTLD mutants demonstrated significant reduction in migration in wound healing assay [99]. Furthermore, they demonstrated that by blocking CTLD with high specificity IgGs, EC migration and tube formation and endothelial cell-to-cell contact was reduced significantly, downstream effects of proposed disruption of CTLD-CTLD interactions and subsequent downregulation of surface-bound CLEC14a in a concentration dependent manner.

In contrast to the notion that the presence of CLEC14a induces endothelial migration, adhesion, and promotion of angiogenesis, Lee et al*.* conducted a series of in vitro experiments concluding that CLEC14a silencing induces angiogenesis leading to hypervascularity of Matrigel plugs [100]. The authors report VEGFR-3 is regulated in parallel with CLEC14a which in turn inversely affects VEGFR-2 signaling, e.g. when CLEC14a is silenced, VEGFR-3 expression and signaling decreases while VEGFR-2 activity compensates, thus leading to increased angiogenesis. The authors do recognize that studies have shown MMRN2 to impair VEGFR-2 signaling, which may have important implications in tumor models as discussed below [101]. Nonetheless, Lee et al*.* gives an alternative perspective of CLEC14a involvement with angiogenesis that warrants further study.

Specific ligand binding is likely responsible for angiogenic effects of CLEC14a. Of particular interest are the binding partners MMRN2 and HSP70-1A. MMRN2, an endothelia-specific protein of the ECM, which also is known to bind CD93 and CD248 [21]. Binding to MMRN2 is dependent on the long loop of the CTLD of not only CLEC14a but CD93 and CD248, yet CD248 binds at a different region than CLEC14a or CD93. To this point, immunohistochemical stains of human PDAC showed colocalization of CLEC14a, MMRN2, and CD248, CLEC14a expression on tumor ECs, CD248 on pericytes engaging tumor endothelium, and MMRN2 spanning between the two. In this study, blocking MMRN2 interaction with ligands, namely CLEC14a, in vitro and in vivo, resulted in angiostatic effect and reduced tumor implant volume, implying that binding MMRN2 with its ligand results in natively proangiogenic signaling, an understanding that is currently debated [101, 102]. It is suggested that MMRN2 acts as “extracellular glue” between CLEC14a-expressing ECs and CD248-positive pericytes/fibroblasts. The mechanisms by which CLEC14a mediates endothelial and ECM remodeling, within MMRN2 binding and beyond, seem to have serious implications for tumor microenvironment status.

Like TM, CLEC14a expression is regulated by shear stress and interacts with HSP70-1A, both of which have potential implications for targeting tumor angiogenesis. About 3% of human genes are known to be regulated by shear stress [103]. In HUVECs, CLEC14a was found to be upregulated ten-fold in static flow compared to laminar shear stress of 2 Pa for 24 h. [94] Co-localization of CLEC14a and known upregulated cell surface marker TIE1 on tumor vessels further suggests CLEC14a expression is regulated by laminar flow. In embryogenesis assays, CLEC14a expression decreased after fetal heartbeat began, implicating pulsatility and increased flows in regulation of CLEC14a. In a similar corollary, atherosclerotic disease, characterized by stenotic blood vessels and thus, decreased flows, shows increased CLEC14a expression proportional to degree of stenosis [104]. Regarding the interaction between CLEC14a and HSP70-1A, in vitro studies by Jang et al*.* demonstrated that HSP70-1A specifically binds the CTLD of CLEC14a and facilitates cell–cell contact by stabilizing CTLD to CTLD adhesion on CLEC14a expressing cells, thus promoting the early steps of angiogenesis. [105]

### CLEC14a: role in malignancy

CLEC14a, along with CD93, is recognized as part of a “common angiogenesis signature” characteristic of primary tumor tissue from head and neck squamous cell, breast, and clear cell carcinomas [30]. Mura et al. demonstrated tumor vessels of breast, prostate, kidney, and thyroid strongly expressed CLEC14a compared to normal vessels [94]. Robinson et al. evaluated CLEC14a expression on healthy samples compared to tumor tissue and confirmed that per EC and normalized to levels of shear stress, tumor tissue exhibited higher levels of CLEC14a such that the normalized CLEC14a levels alone could differentiate healthy vs tumor tissue in 75% of samples [97]. Clear cell renal carcinoma had the highest CLEC14a expression while normal liver and skin samples had higher CLEC14a expression than tumor samples. In contrast, it has been reported that CLEC14a mRNA expression was significantly increased in HCC cells compared to adjacent normal tissue, which could be used as a biomarker of HCC given sensitivity of 85% [106].

In vivo Matrigel plug assays have shown that the addition of blocking CLEC14a mAb to human HCC, PDAC, CRC, and bevacizumab-adapted CRC cells led to significantly decreased MVD and decreased MVD more than bevacizumab treatment in the bevacizumab-resistant CRC cell line [107]. In human glioma xenografts implanted subcutaneously into athymic nude mice, the tumor size was much smaller and rates of apoptosis greater in CLEC14a blocking group on par with bevacizumab-treated group [107]. Similarly, Yan et al*.* found that silencing of CLEC14a in human HCC cell lines, led to higher rates of apoptosis compared to normal liver cells, suggesting a role for CLEC14a in sustaining tumor cell viability [106].

In vivo, Lee et al*.* observed contrasted results of CLEC14a KO [100]. Tumors of KO mice bearing subcutaneously implanted murine tumors exhibited reduced growth rates but significantly shortened overall survival and increased rate of metastases. The authors attributed these results to severely disorganized and immature tumor vasculature demonstrated by lack of pericyte coverage of blood vessels, greater vessel permeability, and higher MVD enabling higher metastatic potential, and thus, worse survival. The authors further showed that in vivo murine tumor models targeted inhibition of VEGFR-2 alleviated the adverse microvasculature alterations and reduced survival seen with CLEC14a only KO. This “rescue” with VEGFR-2 inhibition supports their conclusion that CLEC14a mediates not only VEGF but also receptors of VEGF, further expanding the complex network the CLEC14a, and group XIV proteins interact to positively or negatively mediate angiogenesis.

### CLEC14a: therapeutic approaches

Noy et al. developed an anti-CLEC14a monoclonal antibody (clone C4) that was validated to block the binding of CLEC14a with MMRN2 [108]. In vitro and in vivo assays utilizing specific clone C4, inhibition of CLEC14a-MMRN2 interaction led to inhibition of tube formation, sprouting angiogenesis, and slowed subcutaneous tumor growth. Based on the tumor specificity of CLEC14a demonstrated by human and murine investigation, and the promising results of previous studies knocking out or blocking CLEC14a in tumor progression, Zhuang et al*.* explored the utility of CAR T cell therapy against CLEC14a (Fig. 2d). In three murine cell lines (Rip-Tag, orthotopic PDAC, subcutaneous LLC), the engineered T cell receptor significantly reduced tumor sizes and improved survival rates compared to control mice without undue effects on physiologic wound healing [98]. Total intratumoral vasculature as well as CLEC14a positive structures were significantly reduced while apoptotic markers elevated in CAR treated tumors, suggesting CAR T therapy directed against CLEC14a is cytotoxic and specific. Lastly, nanoparticle delivery of anti-CLEC14a vaccine and siRNA to silence the expression of CLEC14a has recently been explored. One group from the United Kingdom has demonstrated CLEC14a siRNA can be packaged into a chitosan nanoparticle and uptaken preferentially in tumor tissue of subcutaneously implanted LLC tumors in WT mice [109]. Interestingly, the group also demonstrated in vitro studies that nanoparticle delivered CLEC14a siRNA to HUVECs did not alter mRNA expression but rather did so at the protein level. Knockdown of CLEC14a protein resulted in similar changes in endothelial genes seen upon increases in shear stress, suggesting CLEC14a serves a regulatory role of other gene and/or protein expression, rather than itself a 'regulated’ gene, per se. This work highlights angiogenic-specificity of CLEC14a and the complex interplay of CLEC14a among the larger network of endothelia-regulating proteins.

### Group XIV CTLDs, selectins, and galectins

It is worthwhile to consider the group XIV CTLDs within the broader scope of related lectins and their roles in tumorigenesis. Selectins and galectins are two families characterized by conserved carbohydrate binding sites that possess both similarities and deviations in structure and native function to the CTLDs of group XIV [11]. Moreover, much like CD93, CD248, TM, and CLEC14a, selectins and select galectins have been implicated in tumor angiogenesis and metastasis, the mechanisms of which expand the possibilities of study for the CTLDs at hand.

P-, E-, and L-selectins (CD62P, E, L, respectively) are members of the group IV family of CTLDs that share close homology to group XIV CTLDs as type I transmembrane proteins with an *N*-terminal CTLD followed by a single EGF-like domain, variable number of sushi domains, a transmembrane region, and a cytosolic tail [17, 110]. P- and E-selectin, like CD93, TM, and CLEC14a are primarily expressed on ECs, however, unlike group XIV CTLDs, do not directly seem to facilitate angiogenesis but rather mediate activated EC environments by facilitating leukocyte migration, adhesion, and extravasation into tissue [28]. Recognition of surface glycosphingolipids with unique sialylation is key to selectin-ligand binding and subsequent homing of sialylated cells to corresponding signals in physiologic and malignant processes [111]. Clinical and preclinical studies demonstrate epithelial tumor cells possess a high degree of sialylated antigens on their cell surface facilitating several steps of the metastatic cascade such as detachment from the primary tumor, tumor cell aggregation and evasion from immune surveillance in the bloodstream, and extravasation into primed distant organs [111]. Knockout of P- and E-selectins in murine tumor models demonstrate significant abrogation of metastatic disease. [112]

Galectins are another evolutionarily conserved family of glycoproteins with extensive roles in normal physiologic pathways including angiogenesis. Like group XIV CTLDs, galectins contain one or several carbohydrate binding domains that form an anti-parallel beta sheet, however, they lack EGF-like, sushi, and transmembrane domains, the absence of which may enable their prominent role as intracellular transcriptional regulators [113]. Five of the eleven known human galectins, galectin (gal)-1,3,8,9, have been shown to be constitutively produced by ECs and facilitate angiogenesis both in normal and tumor microenvironments [114]. Like CD93, CD248, and CLEC14a, gal-1,3 have been implicated in sustaining VEGF signaling by preventing endocytosis of VEGFR in addition to providing angiostimulatory signals even in the absence of VEGF [115]. In vitro studies have shown that gal-1,3 facilitate EC proliferation, migration, and adhesion to ECM proteins laminin and fibronectin, akin to CD248 and TM [116]. Regarding roles in tumor associated angiogenesis, marked upregulation of gal-1,3 on tumor endothelium has been demonstrated in prostate, lung, colon, and breast cancers [117–120]. Recently, rising serum gal-1 levels in patients with melanoma receiving anti-VEGF therapy have been correlated with drug resistance and worse survival [121]. Genetic ablation of tumor endothelia galectins in murine models of various carcinomas result in decreased intratumoral vessel density, decreased metastases, increased T cell infiltration, and improved survival [122]. Furthermore, like CD248 and CLEC14a, anti-gal-1 vaccine has demonstrated promising anti-tumor effects in preclinical melanoma studies, citing enhanced cytotoxic T cell infiltration within tumors [123]. Distinct from the present CTLDs, galectins possess intracellular roles in promoting tumor growth including activation of focal adhesion kinase (FAK) which modulates integrin expression and facilitates tumor cell migration [124]. Further, one study suggested gal-1 is secreted by tumor cells and endocytosed by tumor ECs to promote H-RAS signaling and EC proliferation. [125]

Taken together, it is clear that selectins, galectins, and group XIV CTLDs share similar origins, yet have evolved unique and distinct mechanisms particularly concerning angiogenesis and endothelial function that drive normal and cancer-associated phenotypes.

### Perspective

Great advances in our understanding of tumor progression and dissemination have been made through breakthroughs in deciphering the tumor vascular environment, in part due to study of the group XIV CTLD family. Such research has borne fruit in novel therapeutics, a select few that have reached human trials. Yet, despite recent progress, there is still much left to be answered.

**Fig. 3:**
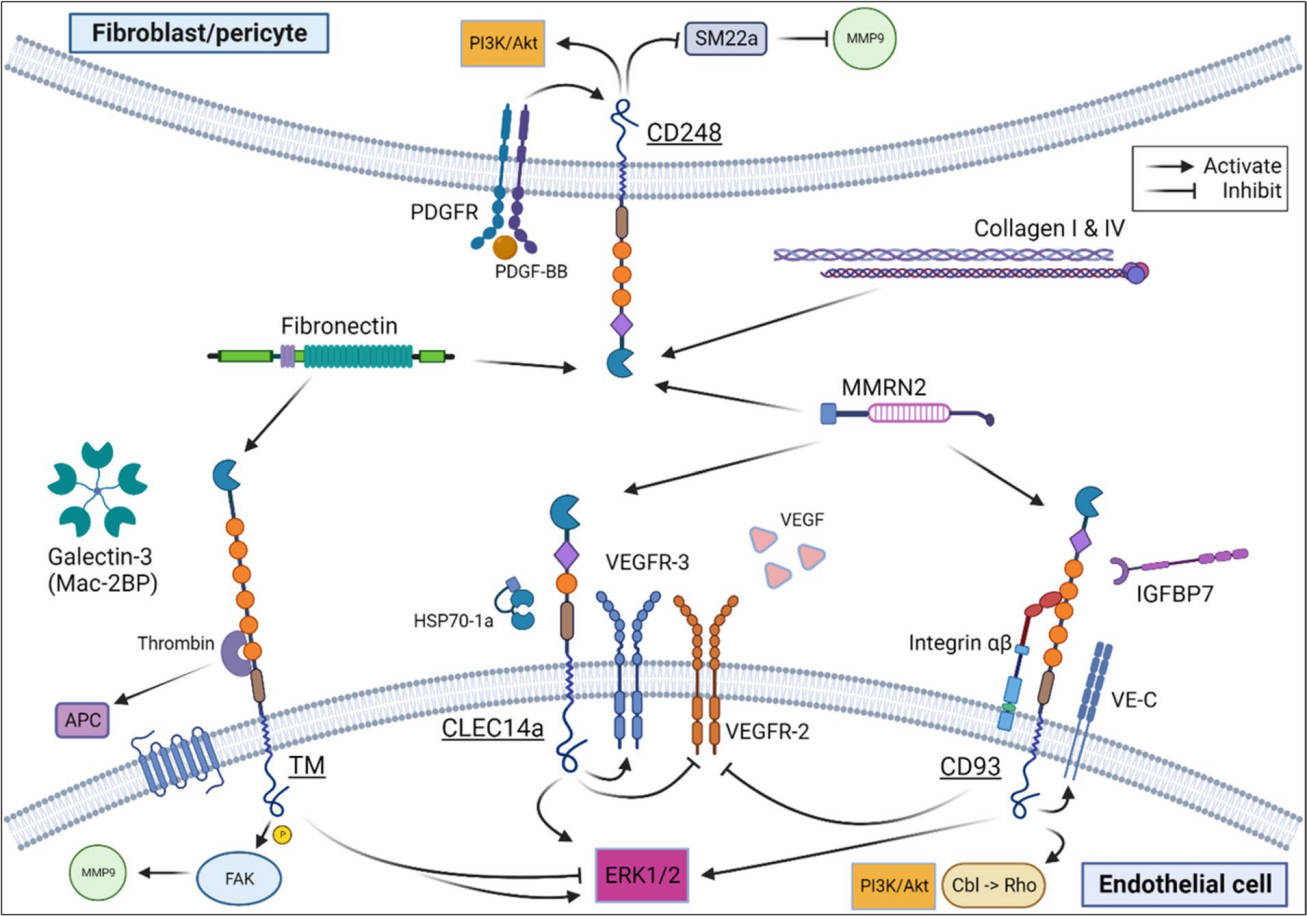
Summary of major interactions of group XIV C-type lectin domain protein family. Thrombomodulin (TM), CLEC14a, and CD93 are predominantly expressed on endothelial cells while CD248 is expressed on stromal cells and structural cells supporting vessels such as fibroblasts and pericytes, respectively. Fibronectin interacts with both TM and CD248 promoting stromal cell migration and adhesion; Multimerin-2 (MMRN2) interacts with CD248, CLEC14a, and CD93 to promote endothelial migration, adhesion, and vessel tube formation; Collagens I and IV interact with CD248 to maintain the extracellular matrix. *FAK* focal adhesion kinase, *MMP9* matrix metalloprotease-9, *APC* activated protein C; *HSP70-1a*, heat shock protein-1a; *VEGF/VEGFR*, vascular endothelial growth factor (receptor), *ERK* extracellular signal-regulated kinase, *PI3k/Akt* phosphoinositide 3-kinase/protein kinase B, *Cbl* Casitas B cell lymphoma protooncogene, *VE-C* VE-cadherin, *IGFBP7* insulin growth factor binding protein 7, *PDGFR* platelet derived growth factor receptor, *PDGF-BB* platelet derived growth factor-BB, *SM22a* transgelin

Regarding the mechanisms by which these group XIV CTLDs function, specifically within the context of the TME, the definitive interactions and pathways leading to endothelial migration, adhesion, remodeling, and ultimately mediation of hematogenous and/or lymphatic spread are yet to be discovered. Specifically, how the interactions between VEGF, VEGFRs, and CLEC14a mediate either pro- or anti-angiogenic state is unclear [100, 101]. While it is known MMRN2 binds CD93, CD248, and CLEC14a, and can bind CD93/IGFBP7 and CD248/CLEC14a to form heterotrimers, the downstream product of these interactions are topics of ongoing investigation [14, 21]. Evidence suggesting CD248 expression on naïve T cells is another potential avenue for further study, as CD248 expression on T cells could be a means of tumor-driven immunosuppression, and therefore a potential novel target for immunotherapy [48]. How TM executes a metastasis-prohibiting role within the larger context of a complex coagulation network has substantial potential to unify several unknown physiologic and pathologic processes alike. A graphic summary of group XIV CTLD major binding interactions and downstream signaling pathways are displayed in Fig. 3.

To achieve clinical success in human trials, investigation into discrepancies within preclinical models and their results is warranted. Site specificity regarding response to CD248 knockdown in murine tumor implantation has been reported, citing lack of anti-tumor response in subcutaneously implanted tumors compared to abdominally implanted ones. Yet, other studies such as that from Rybinski et al. report the response of subcutaneously implanted xenografts to CD248 blockade also favorable, which is potentially due to human versus mouse origins of tumors and CD248 [46, 61]. Furthermore, discrepancies in the downstream effects of CLEC14a KO in in vivo models also exists – one study citing clearly higher metastases and survival in KO mice compared to controls and other studies citing the opposite [99, 100, 107, 108]. Thorough investigation and comparison of experimental design could ultimately lead to the discovery of new mechanisms connecting these varying results, and thus offer a potential new therapeutic target.

The last few decades have ushered in a whirlwind of novel and evolving therapies such as CAR T cell, adoptive T cell transfer, targeted monoclonal antibody blockade, antibody–drug conjugates, nanoparticle delivery, small molecule inhibitors, and anti-cancer vaccines. With the success of immune checkpoint inhibitors and anti-VEGF mAbs in the clinic, highly specific antibodies targeting a specific ligand have been at the forefront of immunotherapy. MAbs against CD248 and CD93 have shown promise in the preclinical setting and been/are testing in clinical trials, although the former with limited success. Nonetheless, future investigation into optimizing the delivery and specificity, and the elucidation of downstream function of these highly specific antibodies is warranted. Future research should also be directed to exploring small molecule inhibitors as a reliable method of neutralizing effector CTLD binding domains. Lastly, given that group XIV proteins act in concert with an abundance of other ligands in a complex network of signaling pathways supplying physiologic and malignant angiogenesis, it may be of synergistic benefit to explore combined inhibition of these proteins and their pathways. A summary of preclinical and clinical applications targeting group XIV CTLDs is presented in Table 1.

Considering group XIV CTLDs in the context of other lectins such as selectins and galectins offers potential avenues of study to expand our knowledge of the mechanisms and thereby targets responsible for tumor vascularization and growth. Given the structural similarity between group XIV CTLDs and selectins, and the documented cell–cell adhesion properties of CD93, it is plausible that group XIV proteins can likewise directly facilitate circulating tumor cell adhesion and intravasation. Further, upregulation of selectins and CD93, CD248, and CLEC14a on tumor ECs prompt investigation as to whether group XIV CTLDs can serve as a mediator or master regulator of the activated EC phenotype in the face of tumor-derived signals as is described for E-selectin. Regarding galectins, their prominent intracellular roles mediating transcriptional activity via glycan binding suggest that CTLDs could carry out similar actions. Lastly, while beyond the scope of this review, tumor-driven immunosuppressive mechanisms utilizing selectin and galectin ligand interactions warrant examination of similar adaptations applied to group XIV CTLDs.

A final comment is reserved for consideration of the general approach to targeting tumor angiogenesis. Current immunotherapies targeting tumor vessels and their development predominantly emphasize the abrogation and depletion of abnormal tumor vessels, in effort to ‘cutoff’ blood supply, and therefore nutrients, to an expanding lesion. However, the concept of vessel normalization has risen in the last few years as a potentially more sustainable and efficacious approach to targeting tumor angiogenesis. Several reports suggest that even the current commercially available immunotherapies normalize tumor vessels, albeit for a transient length of time [16, 126]. Nonetheless, normalization of tumor vasculature has been found to promote a host of favorable alterations to the TME, as we have recently demonstrated in preclinical murine models by disrupting CD93 interaction with IGFBP7/MMRN2 [14]. The contrast between CD93 blockade with our mAb leading to tumor vessel normalization compared to companion investigations of group XIV protein KO leading to increased MVD and dysfunctional vessels within tumors in the absence of their CTLD protein, is pronounced. Accumulating evidence suggests a role of antibody composition, namely crystallizable fragment (Fc) inclusion and triggering of antibody-dependent cell-mediated cytotoxicity (ADCC), to potentially mediate depleting versus normalizing pathways [127]. Further investigation into what mechanisms drive depletion or normalization is underway. Furthermore, it would be advantageous to understand which tumors respond best to which angiogenic approach, thus maximizing therapeutic benefit. A deeper understanding of group XIV CTLDs role in tumorigenesis may help bridge these knowledge gaps.

## Conclusion

Group XIV CTLDs play pivotal roles in a variety of physiologic functions from endothelial and ECM structure, organization, and function to maintaining coagulative balance. Their native expression and activity are severely dysregulated in cancer and contribute to formation of hostile tumor microenvironments. Each protein possesses its own unique features and specificity yet exists in a complex and diverse network of binding partners and downstream signaling pathways. Effectively targeting tumor angiogenesis remains an elusive feat that an evolving understanding of group XIV CTLDs may help us obtain.

## Data Availability

Not applicable.

## Abbreviations

CTLD: C-type lectin domain
VEGF: Vascular endothelial growth factor
EGF: Epidermal growth factor
ECM: Extracellular matrix
MMRN2: Multimerin-2
HSP70-1: Heat shock protein 70-1
TME: Tumor microenvironment
CRC: Colorectal cancer
EC: Endothelial cell
C1qRp: C1q receptor
IGFBP7: Insulin growth factor binding protein 7
mAb: Monoclonal antibody
HUVEC: Human umbilical vein endothelial cell
HDMEC: Human dermal microvascular endothelial cell
TEM1: Tumor endothelial marker 1
CHO: Chinese hamster ovary
MMP: Matrix metalloprotease
SM22a: Transgelin
TGF-β: Transforming growth factor beta
WT: Wildtype
PDGF-BB: Platelet derived growth factor BB
SAGE: Serial analysis of gene expression
αSMA: Alpha smooth muscle actin
NG2: Neural/glial antigen 2
IHC: Immunohistochemistry
OS: Osteosarcoma
LLC: Lewis lung carcinoma
MVD: Microvessel density
HCC: Hepatocellular carcinoma
ADC: Antibody drug conjugate
CAR T: Chimeric antigen receptor T cell
MMAE: Monomethyl auristatin E
BiTE: Bispecific T cell engager
TM: Thrombomodulin
PAR: Protease activated receptor
TAFI: Thrombin activatable fibrinolysis inhibitor
GPCR: G coupled protein receptor
SCC: Squamous cell carcinoma
PDAC: Pancreatic ductal adenocarcinoma
EMT: Epithelial to mesenchymal transition
NET: Neutrophil extracellular trap
DC: Dendritic cell
Th1: T helper cell subset 1
HMGB: High mobility group box
ADCC: Antibody-dependent cellular cytotoxicity
Fc: Fragment crystallizable
APC: Activated protein C
